# Alpha-synuclein alters differently gene expression of Sirts, PARPs and other stress response proteins: implications for neurodegenerative disorders

**DOI:** 10.1007/s12035-016-0317-1

**Published:** 2017-01-03

**Authors:** J. Motyl, P. L. Wencel, M. Cieślik, R. P. Strosznajder, J. B. Strosznajder

**Affiliations:** 1 Department of Cellular Signalling, Mossakowski Medical Research Centre, Polish Academy of Sciences, 5 Pawińskiego Street, Warsaw, Poland; 2 Laboratory of Preclinical Research and Environmental Agents, Department of Neurosurgery, Mossakowski Medical Research Centre, Polish Academy of Sciences, 5 Pawińskiego Street, 02-106 Warsaw, Poland

**Keywords:** Alpha-synuclein, Sirtuins, PARPs, Amyloid, Neurodegeneration, AD

## Abstract

Alpha-synuclein (ASN) is a presynaptic protein that can easily change its conformation under different types of stress. It’s assumed that ASN plays an important role in the pathogenesis of Parkinson’s and Alzheimer’s disease. However, the molecular mechanism of ASN toxicity has not been elucidated. This study focused on the role of extracellular ASN (eASN) in regulation of transcription of sirtuins (Sirts) and DNA-bound poly(ADP-ribose) polymerases (PARPs) - proteins crucial for cells’ survival/death. Our results indicate that eASN enhanced the free radicals level, decreased mitochondria membrane potential, cells viability and activated cells’ death. Concomitantly eASN activated expression of antioxidative proteins (*Sod2*, G*px4*, *Gadd45b)* and DNA-bound *Parp2* and *Parp3.* Moreover, eASN upregulated expression of *Sirt3* and *Sirt5*, but downregulated of *Sirt1*, which plays an important role in cell metabolism including Aβ precursor protein (APP) processing. eASN downregulated gene expression of APP alpha secretase (*Adam10*) and metalloproteinases *Mmp2, Mmp10* but upregulated *Mmp11.* Additionally, expression and activity of pro-survival sphingosine kinase 1 (Sphk1), Akt kinase and anti-apoptotic protein *Bcl2* were inhibited*.* Moreover, higher expression of pro-apoptotic protein *Bax* and enhancement of apoptotic cells’ death were observed. Summarizing, eASN significantly modulates transcription of *Sirts* and enzymes involved in APP/Aβ metabolism and through these mechanisms eASN toxicity may be enhanced. The inhibition of Sphk1 and Akt by eASN may lead to disturbances of survival pathways. These results suggest that eASN through alteration of transcription and by inhibition of pro-survival kinases may play important pathogenic role in neurodegenerative disorders.

**Electronic supplementary material:**

The online version of this article (doi:10.1007/s12035-016-0317-1) contains supplementary material, which is available to authorized users.

## Introduction

Alpha-synuclein (ASN) is a 140-amino acid soluble protein that is abundantly expressed in the nervous system, where it constitutes 1% of total cytosolic proteins [1–3]. In physiological conditions, ASN occurs in presynaptic terminals in close proximity to synaptic vesicles. ASN is involved in the regulation of synaptic vesicle transport and in the formation of synaptic connections, their structure and plasticity [4–7]. The data of Bartels et al. 2011 [8] indicate that ASN occurs physiologically as a helically folded tetramer that is resistant to aggregation. The tetramer can dissolve into unfolded monomers which subsequently can aggregate into soluble protofibrils and insoluble β-amyloid fibres [9]. Recent data have indicated that alterations in ASN expression and conformation could play an important role in familial (A30P, A53T mutations) and in sporadic forms of Parkinson’s disease (PD) as well as in the pathology of about 60% of Alzheimer’s disease’s (AD) cases [10–13]. Misfolding of this protein leads to aggregation/ fibrilisation of ASN, which in β-sheet structure is toxic to cells [14–16]. The aggregates of ASN are the main components of intracellular inclusions called Lewy bodies (LBs), which are the pathological hallmarks of PD, AD-forms with LBs and other synucleinopathies [17–21]. The latest studies including our data demonstrate that ASN could be secreted from neuronal cells and nerve endings into the extracellular space [12, 22, 23]. Extracellular alpha-synuclein (eASN) can alter ionic homeostasis and synaptic transmission in neuronal cells [24, 25]. Several recent studies support the hypothesis that, just as the human prion protein, ASN can transfer protein alteration from cell to cell [26, 27]. Recently, ASN was detected in rodent and human brain interstitial fluid, which confirms that it is secreted outside the cell. eASN affects neuronal and glial homeostasis, activates inflammatory reactions and promotes neuronal death [12, 28–32]. Moreover, eASN induces amyloid-beta (Aβ) secretion and enhances the level of the amyloid-beta precursor protein (APP), and in this way it potentiates its own and Aβ toxicity [11, 23, 27, 33–36]. The mechanism of ASN secretion is not well understood, however, oxidative stress seems to have a promoting role in this process [12, 22, 29].

Our last study indicated that ASN secretion is also modulated by the pharmacological inhibition of sphingosine kinase(s) (Sphk1/2) [22] and this effect is probably mediated by free radical–dependent processes. These enzymes are responsible for the synthesis of sphingosine-1-phosphate (S1P), a pleiotropic lipid mediator which exerts a mitogenic, pro-survival but also pro-apoptotic effects within the cell [37–40]. Sphk1/2 are key enzymes that maintain homeostasis between S1P and ceramide, and through this mechanism they may play an important role in the regulation of cell survival and death. The inhibition of Sphk1/2 alters S1P-dependent signalling, regulated also by the PI3K/Akt pathway. The three from five receptors (S1P1, S1P2 and S1P3) are specific for S1P transduce information by PI3K/Akt. Our last data indicated the neuroprotective effect of S1P (1μM) in dopaminergic cells-exposed to different types of stress [41–43]. The lower S1P concentration has been described in AD [40, 44], in the dopaminergic SH-SY5Y cell PD model and also in the animal PD model evoked by 1-methyl-4-phenylpyridinium MPP+/MPTP, respectively [22, 41, 45]. The alteration of S1P level in AD correlated well with reduced expression/activity of Sphk1/2 and with the ratio of dementia.

Another important role in regulation of cell viability is played by nicotinamide adenine dinucleotide (NAD) dependent enzymes such as sirtuins (SIRTs) and DNA-bound poly(ADP-ribose) polymerases (PARPs). The enzyme families of SIRTs and PARPS are engaged in the regulation of energy metabolism, anti-oxidative processes, DNA repair and cell survival [46–49]. In mammalian cells, there are seven members of the sirtuins family (SIRTs 1-7), among which SIRT1 has been the most investigated. Recently, it was found that SIRT1 protects cells against ASN and protein Tau aggregation. The lifespan of mouse is increased by overexpressing SIRT1 and decreased by knocking out SIRT1 in brain [50–52]. SIRT1 activates alpha-secretase gene expression (*Adam10*) and supresses amyloid beta (Aβ) production [53]. Alpha-secretase activates APP processing inside the Aβ sequence and in this way prevents formation of neurotoxic Aβ. Degradation of APP by alpha-secretase leads to release of soluble, neuroprotective terminal domain of APP. Several metalloproteinases as ADAM10, ADAM17, ADAM9 express alpha-secretase activity [54]. Moreover, SIRT1 activates peroxisome proliferator-activated receptor (PPAR)γ coactivator 1α (PGC1α) and through this mechanism it increases mitochondrial biogenesis [47]. Among mitochondrial located SIRTs, SIRT3 was the best investigated and it was demonstrated that this enzyme is responsible for the regulation of electron transport protein complexes (ETC) and for expression and activity of several anti-oxidative proteins, e.g. superoxide dismutase (SOD2) and glutathione peroxidase (GPx), which are crucial in the molecular mechanism of cell viability [46]. The roles of other mitochondrial SIRTs , SIRT4 and SIRT 5 is not fully understood. Outeiro et al. [55] found that inhibition of cytosolic SIRT2 protects against ASN toxicity in vitro and in the Drosophila model of PD. It was indicated that this cytosolic-located SIRT2 exerted the opposite effect than pro-survival SIRT1 [49]. The other NAD-regulated enzyme family (17 members) of PARPs, as compared to SIRTs, exhibits higher affinity to the βNAD^+^ particle [56, 57]. The most important enzyme of this family is DNA-bound PARP1, which in the brain is responsible for more than 90% of poly-ADP-ribosylation processes [58, 59]. Also PARP2 and PARP3 are DNA-bound enzymes, and all of them are activated in stress and are involved in the DNA repair mechanism under middle stress [60]. However, under massive DNA damage, PARP1 can be over-activated and responsible for apoptotic or necrotic cell death [58, 61, 62].

In this study we investigated the role of eASN in the regulation of gene expression of SIRTs, PARPs and enzymes involved in the APP/Aβ metabolism. Moreover, the expression and activity of Sphk1 and Akt under eASN toxicity were analysed.

## Materials & Methods

### Aggregation of a-synuclein

The ASN protein was subjected to the aggregation/oligomerisation procedure as described in Danzer et al. [33] with some modifications. Lyophilised ASN (from rPeptide, USA) was dissolved in 1 ml mixture of 50 mM sodium phosphate buffer, pH 7.0, containing 20% ethanol, to a final concentration of ASN 10 μM. After 4 h of shaking at room temperature (RT) using a thermomixer 5436; Eppendorf, Wesseling-Berzdorf, Germany), the ASN protein was lyophilised again and resuspended in 0.5 ml mixture of 50 mM sodium phosphate buffer, pH 7.0, containing 10% ethanol. Then it was mixed for 24 h with open lids to evaporate the residual ethanol. Concentrations of obtained ASN forms were determined using spectrophotometric measurement (NanoDrop) with absorbance at 280/290 nm.

### Verification of ASN Purity and Structure

The purity of the ASN protein was determined using mass spectrometry/HPLC. Then aliquots containing 2 μg of the ASN protein prepared after the procedure as described above (Danzer et al. [33]) were analysed by SDS-PAGE followed by silver staining. The analysis indicated that ASN before and after the described procedure was in monomeric/oligomeric form. Then the ASN pure protein before and after the aggregation/oligomerisation procedure was analysed by circular dichroism (CD) on a JASCO J-815 CD spectropolarimeter in the range of ~270-195 nm with a data pitch of 1.0 nm. ASN before the procedure was in a random coil structure which was no longer observed after the aggregation/oligomerisation procedure. This indicated that the structure of ASN changed into the β-sheet structure. In addition, the conformation state of ASN was confirmed using Thioflavin T (ThT, benzothiazole dye) fluorescence.

### Cell Culture and Cell Treatment Protocol

Rat *pheochromocytoma* (PC12) cells were cultured in Dulbecco’s Modified Eagle’s Medium (DMEM) supplemented with 10% heat-inactivated fetal bovine serum (FBS), 5% heat inactivated horse serum, 2 mM L-glutamine, 50 U/ml penicillin and 50 μg/ml streptomycin in a 5% CO2 atmosphere at 37 °C. Cell treatment was performed in low-serum (2% FBS) DMEM to stop proliferation. The PC12 cells were used for experiments between five and ten passage numbers. For the MTT assay, the PC12 cells were seeded onto collagen-coated 96-well plates at a density of 7×10^4^ cells per well in 100 μl of medium. For other analyses, the PC12 cells were seeded at 3×10^5^ cells/10-mm tissue culture dishes. Then the PC12 cells were treated with eASN (0.5 μM for 24-48 h). Control cells were treated with sodium phosphate buffer subjected to the same oligomerisation procedure as the eASN. Additionally, cells were treated with Z-DEVD-FMK (R&D Systems), Cyclosporin A (Sigma-Aldrich, 30024), SEW2871 (Cayman Chemical), p-FTY720 (Cayman Chemical), AK-7 (Sigma-Aldrich, SML0152), PJ-34 (Sigma-Aldrich), Resveratrol (Sigma-Aldrich), Quercetin (Sigma-Aldrich). Appropriate solvent was added to respective controls.

### Cytotoxicity Assays

#### Cell Viability Analysis (MTT Assay)

Mitochondrial function and cellular viability were evaluated using 2-(4,5-dimethylthiazol-2-yl)-2,5-diphenyltetrazolium bromide (MTT). After 48 h incubation with the appropriate compounds, MTT (2.5 mg/ml) was added to all of the wells. The cells were incubated at 37 °C for 2 h. Then the medium was removed, the formazan crystals were dissolved in DMSO and absorbance at 595 nm was measured.

#### Trypan Blue Staining

Trypan blue solution was added to the culture medium. The cells were examined immediately under an optical microscope. The number of blue stained cells and the total number of cells were counted. If cells took up trypan blue, they were considered nonviable.

#### Determination of Apoptosis Using Hoechst 33342 Fluorescent Staining

For morphological studies, PC12 cells were subjected for 24-96 h to oxidative stress evoked by eASN (0,5 μM). PC12 cells were collected and washed in PBS. The cells were fixed in MetOH for 30 min in 4 C. Nuclei were visualised with Hoechst 33342 (0.2 μg/ml, Riedel-de-Haën Germany) fluorescent staining. The cells were examined under a fluorescence microscope (Olympus BX51, Japan) and photographed with a digital camera (Olympus DP70, Japan). Cells with typical apoptotic nuclear morphology (nuclear shrinkage, condensation) were identified and counted. The results were expressed as apoptotic index according to the equation apoptotic index=(apoptotic ratio/average apoptotic ratio for control) where apoptotic ratio=(apoptotic cells )/(all cells).

#### Mitochondrial membrane potential (ΔΨm) assay

Detection of mitochondrial membrane potential (ΔΨm) was performed using the JC-1 detection kit (Thermo Fisher Scientific) according to the manufacturer’s directions. JC-1 (5′,6,6′-tetrachloro-1,1′,3,3′-tetraethylbenzimidazolylcarbocyanine iodide) is a cationic dye which accumulates in mitochondrial membranes of healthy cells, resulting in red fluorescence (590 nm), while in apoptotic and necrotic cells, which have diminished mitochondrial membrane potential, JC-1 exists in the green fluorescent (529 nm) monomer form. Images are captured using a fluorescence image scanning unit (FMBIO III) instrument (flow cytometer) and the ratios of red (live cells) and green (dead cells) fluorescence were calculated. All assays were performed in quadruples and repeated twice.

#### Determination of Free Radicals Using 2’7’-dichlorofluorescein (DCF)

The level of reactive oxygen species (ROS) was determined using 2’,7’ dichlorodihydrofluorescein diacetate (H2DCF-DA) exactly as described previously by Cieślik et al. 2015 [63].

#### Determination of Sphk1 Activity

Sphingosine kinase activity assay was performed according to the method of Don et al. 2007 [64], as described previously [22, 41]. After 24 h incubation, the PC12 cells were washed with iced PBS and lysed in 50 mM Hepes, pH 7.4, 15 mM MgCl_2_, 10 mM KCl,10% glycerol, 2 mM ATP, 5 mM NaF, 1 mM deoxypyridoxine, and EDTA-free complete protease inhibitor (Roche Applied Science). Lysates were cleared by centrifugation at 15 000 g for 5 min. The 100 μg of lysates and NBD-Sphingosine (10 μM final) (Avanti Polar Lipids) were mixed in reaction buffer, 50 mM Hepes, pH 7.4, 15 mM MgCl_2_, 0.5% Triton X-100, 10% glycerol, 2 mM ATP and incubated for 30 min at 30 °C. The reactions were stopped by the addition of an equal amount of 1 M potassium phosphate (pH 8.5), followed by the addition of 2.5-fold chloroform/methanol (2:1), and then centrifuged at 15 000 g for 1 min. Only the reactant NBD-S1P, but not the substrate NBD-sphingosine, was collected in the alkaline aqueous phase. After the addition of an equal volume of dimethylformamide, the fluorescence value was determined (λex = 485 nm, λem = 538 nm).

#### Immunochemical Determination of Protein Level (Western Blot)

The PC12 cells were washed three times with ice-cold PBS, scraped from the culture dishes and suspended in 1x Cell Lysis Buffer (from Cell Signalling Technology). Protein levels were determined using the Lowry method [65], and the proteins were mixed with 5× Laemmli sample buffer and denatured for 5 min at 95 °C. A total of 50 μg of the protein was loaded per lane on 7.5%, 10% or 15% acrylamide gels and separated by sodium dodecyl sulfate (SDS)-polyacrylamide gel electrophoresis. The proteins were transferred onto a nitrocellulose membrane at 10V overnight at 4 °C. The quality of transfer was verified with Ponceau S staining. The membranes were incubated in 5% dry milk in TBS with Tween 20 (TBS-T) for 1 h at RT and exposed overnight at 4 °C to the following antibodies: anti-Sphk1 (Cell Signalling Technology, 1:500), anti-pAkt and anti-Akt (Cell Signalling Technology, at a dilution of 1:1000), anti-SIRT1 (Santa Cruz Biotechnology, 1:1000) and anti-Gapdh (Sigma-Aldrich, 1:50 000). After treatment for 1 h with the corresponding horseradish peroxidase-coupled secondary antibodies (anti-rabbit from Amersham Biosciences or anti-mouse from GE Healthcare), the protein bands were detected by chemiluminescent reaction using ECL reagent (Amersham Biosciences). GAPDH was detected on membranes as a loading control. Densitometric analysis and size-marker-based verification were performed using Total Lab 4 software. After detection, the membranes were treated with stripping buffer (50 mM glycine, pH 2.5, 1% SDS) for further blots.

#### Determination of Gene Expression

The PC12 cells were washed twice with ice-cold PBS and suspended in 1 ml of TRI reagent (Sigma-Aldrich). RNA was isolated from the cell pellet according to the manufacturer’s protocol. Digestion of DNA contamination was performed by using DNase I according to the manufacturer’s protocol (Sigma-Aldrich). Reverse transcription was performed using a High Capacity cDNA Reverse Transcription Kit according to the manufacturer’s protocol (Applied Biosystems, Foster City, CA, USA). The level of mRNA for selected genes was analysed using TaqMan Gene Expression Assays (Applied Biosystems, Foster City, CA, USA) according to the manufacturer’s instructions: *Bax*: Rn01480161_g1, *Bcl2*: Rn99999125_m1, *Bcl2l1*: Rn00437783_m1, *Adam10*: Rn01530753_m1, *Bace1*: Rn00569988_m1, *Psen1*: Rn00569763_m1, *Psen2*: Rn00579412_m1, *Sod1*: Rn00566938_m1, *Sod2*: Rn00690588_g1, *Cyb5b*: Rn00577982_m1, *Gadd45b*: Rn01452530_g1, *Gpx4*: Rn00820818_g1, *Sirt1:* Rn01428096_m1*, Sirt2:* Rn01457502_m1*, Sirt3*:Rn01501410_m1, *Sirt4*: Rn01481485_m1, *Sirt5*:Rn01450559_m1, *Parp1*: Rn00565018_m1, *Parp2*: Rn01414610_m1, *Parp3*: Rn01447502_m1, *Mmp2*: Rn01538170_m1, *Mmp9*: Rn00579162_m1, *Mmp10*: Rn00591678_m1, *Mmp11*: Rn01428817_m1, *Actb*: 4352340E. *Actb* was selected and used in all of the studies as a reference gene. Quantitative PCR was performed on an ABI PRISM 7500 apparatus. The relative levels of mRNA were calculated using the ΔΔCt method.

#### Statistical Analysis

The results were expressed as mean values ± SEM. Differences between the means were analysed using a Student’s t-test for two groups or one-way analysis of variance ANOVA with the Newman–Keuls post hoc test among multiple groups, p values < 0.05 were considered significant. The statistical analyses were performed using Graph Pad Prism version 5.0 (Graph Pad Software, San Diego, CA, USA).

## Results

**Fig. 1.**
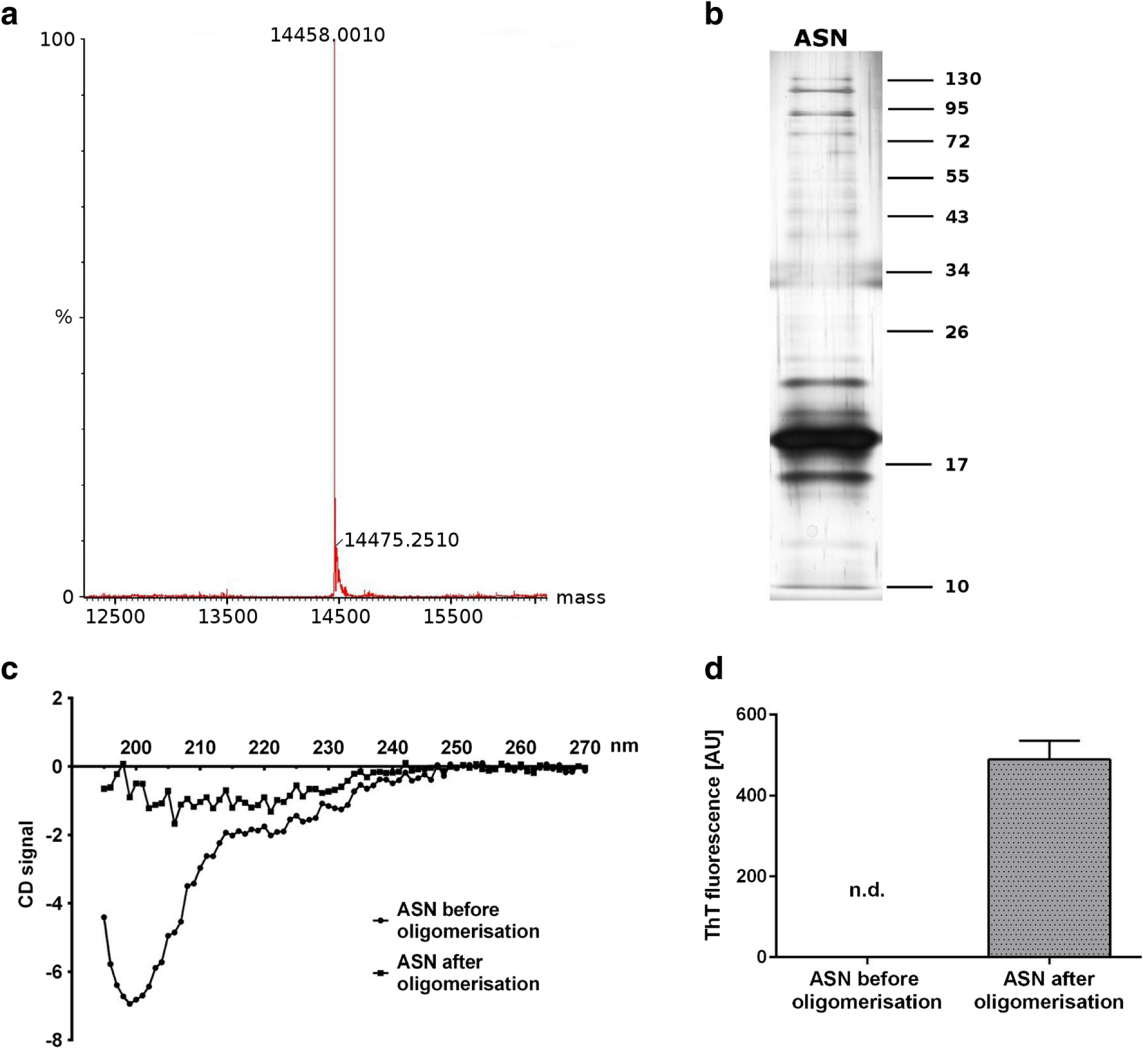
Determination of eASN purity and structure. eASN used for aggregation /oligomerisation procedure (A/O) was subjected to MMS/HPLC analysis of its purity in 50 mM sodium phosphate buffer, pH 7.0 before and after the A/O procedure **(a)**. Then the electrophoretic analysis of the eASN aggregation forms was performed. 2 μg of protein before and after the A/O procedure was subjected to non-denaturing electrophoresis followed by silver staining **(b)**. The presence of eASN monomers, dimers and trimers was reported. In the next step eASN before and after the A/O procedure was subjected to analysis of circular dichroism spectra of eASN in 50 mM sodium phosphate buffer, pH 7.0 **(c)**. Note the significant differences in spectra before and after eASN oligomerisation procedure. Finally, analysis of Thioflavin T(ThT) fluorescence before and after eASN oligomerisation was done (**d**)

In the present research, we studied the molecular mechanism of eASN evoked cytotoxicity leading to a cells’ death. The study was focused on the role of eASN in regulation of gene expression of sirtuins, DNA-bound PARPs and other stress response proteins engaged in regulation of cell survival/death. The MSS/HPLC analysis of ASN used in this study indicated its purity (Fig. 1a). It was found that ASN which was used for the experiments, adopted the monomeric/oligomeric forms (Fig. 1b). Using circular dichroism (CD) it was observed that ASN was in random coil structure (Fig. 1c), which changed during the aggregation/oligomerization procedure into the β-sheet structure - confirmed by thioflavin T fluorescence determination (Fig. 1d).

**Fig. 2.**
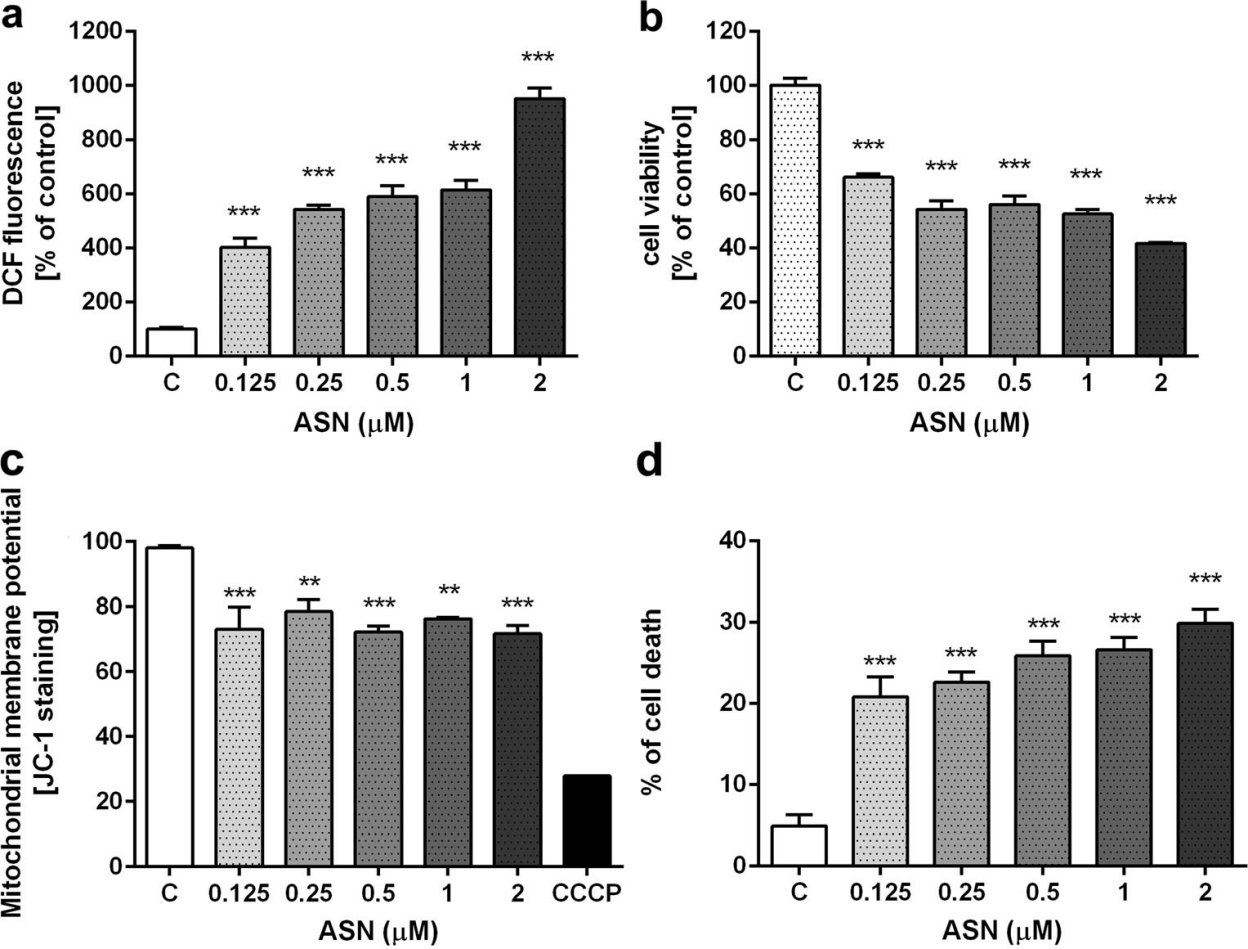
The effect of eASN on ROS generation, PC12 cells’ viability, mitochondrial membrane potential and cells’ death. PC12 cells were treated with 0,125–2 μM eASN for 48 h. ROS generation was determined using DCF probe **(a),** cell viability by MTT assay **(b)**, mitochondrial membrane potential determined by JC-1 staining **(c)**, cells’ death by Trypan Blue staining **(d)**. Data represent the mean value ± S.E.M of four-six independent experiments with four to six replications. *p<0.05, **p<0.01 and ***p<0.001 versus control (phosphate buffer - treated PC12 cells) by one-way ANOVA followed by the Newman–Keuls post-hoc test.

This study demonstrated that exogenous, extracellularly applied eASN in monomeric/oligomeric form significantly enhanced the free radicals level in a concentration-dependent manner (Fig. 2a) and concomitantly reduced PC12 cells’ viability (Fig. 2b). About 50% of cells show low viability at 0.5 μM of eASN and this concentration was further used. For analysing the effect of eASN on cells’ viability, the mitochondrial membrane potential (MMP) using JC-1 staining was evaluated. eASN significantly decreased MMP by about 20% comparing to the control cells (without eASN) (Fig. 2c). Experiments with trypan blue staining demonstrated a significant increase in number of dead cells under the eASN toxicity conditions (Fig. 2d).

**Fig. 3.**
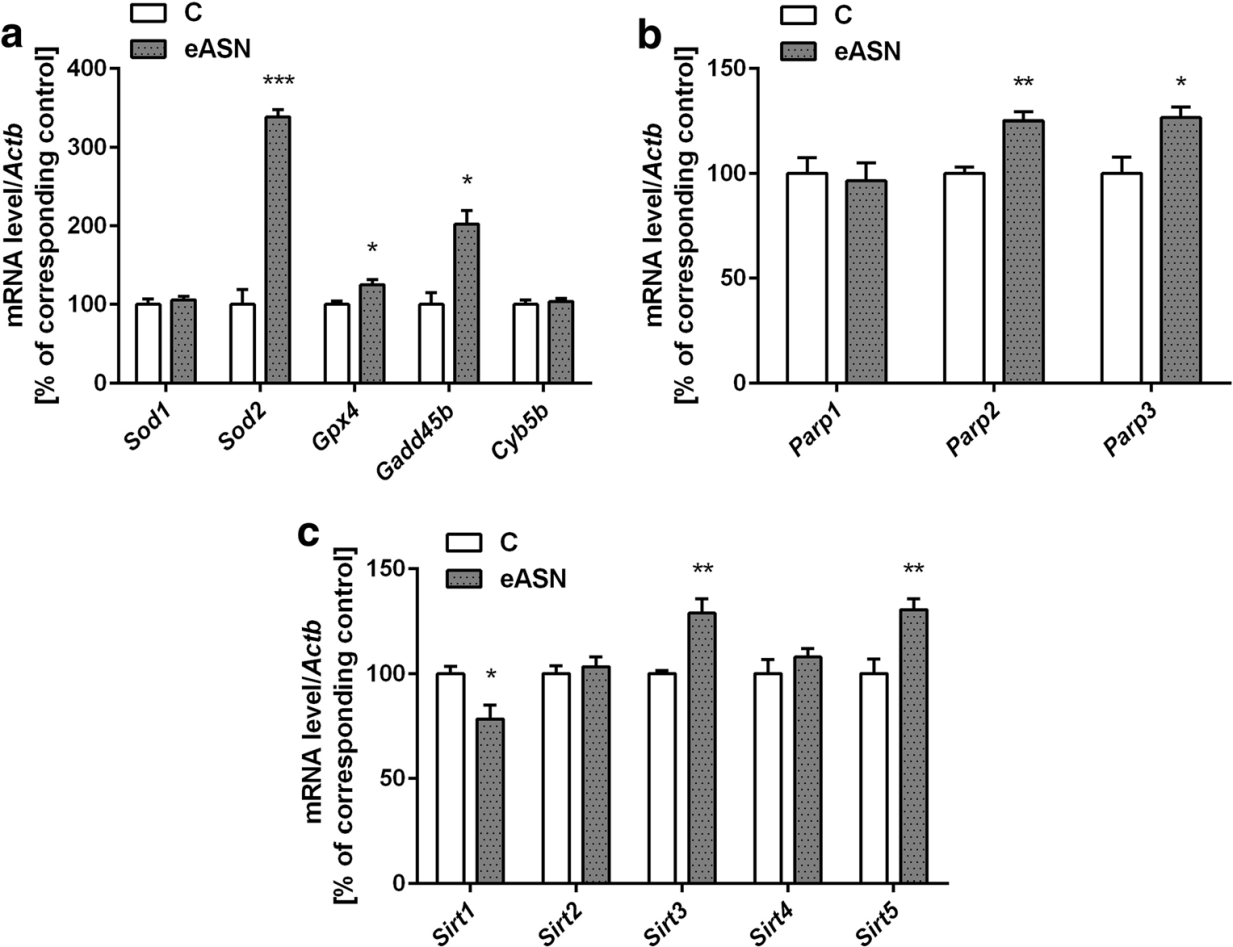
The effect of eASN on gene expression of anti-oxidative enzymes and DNA-bound PARPs. The mRNA level of *Sod1*, *Sod2*, *Gpx4*, *Gadd45b*, *Cyb5b* **(a)**, *Parp1,2,3* **(b)**, *Sirt1,2,3,4,5* **(c)** after 24 h of 0,5 μM eASN treatment was measured with real-time PCR. The value expresses the fold of the above gene stimulation normalized against *Actb* (β-actin). Data represent the mean value ±S.E.M of four separate experiments. The relative level of mRNA was calculated by ΔΔCt method. *p<0.05, and ***p<0.001 versus control (phosphate buffer -treated PC12 cells) by Student’s t-test.

The eASN evoked stress may lead to activation of cytoprotective processes to counteract free radicals mediated damages of macromolecules. We determined the transcription level of selected enzymes involved in antioxidative defence against eASN toxicity. eASN significantly increased the mRNA level of the mitochondrial anti-oxidative enzymes: superoxide dismutase 2 (*Sod 2*), glutathione peroxidase 4 (*Gpx4*) as well as *Gadd45b* (anti-apoptotic protein growth arrest and the DNA-damage-inducible beta) (Fig. 3a). There was no significant effect of eASN on *Sod1* and cytochrome b5 (*Cyb5b*) gene expression (Fig. 3a). Moreover, DNA-bound PARPs expression was determined under eASN evoked oxidative stress. Gene expression of *Parp1* was not altered by eASN, but *Parp2* and *Parp3* were significantly upregulated (Fig. 3b). The protein level of the mitochondrial apoptosis-inducing factor (AIF) regulated by PARP/PAR was not changed as compared to the control conditions (data not shown). The recent studies demonstrated the significant role of other NAD dependent enzymes sirtuins (SIRTs) in the regulation of anti-oxidative defence in cells. Our results showed that mRNA level of *Sirt3* and *Sirt5* (mitochondria located enzymes) was significantly enhanced, but expression of *Sirt1* was significantly decreased and *Sirt2, 4* were not altered (Fig. 3c).

**Fig. 4.**
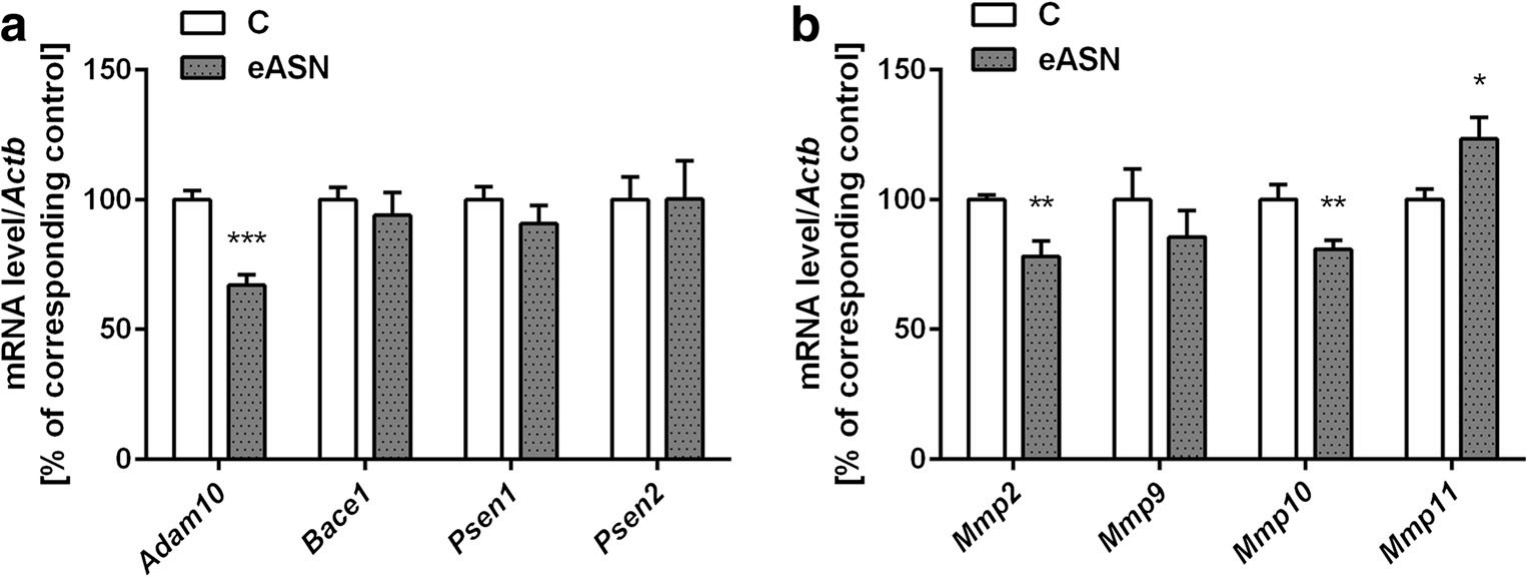
Effect of eASN on gene expression of selected Aβ secretases and metalloproteinases. The mRNA level of *Adam10*, *Bace1, Psen1*, *Psen2* **(a)** and *Mmp2,9,10,11* ***(b)*** after 24 h of 0,5 μM eASN treatment was measured with real-time PCR. The value expresses the fold of the above gene stimulation normalized against *Actb* (β-actin). Data represent the mean value ± S.E.M of four-six separate experiments with four replications. The relative level of mRNA was calculated by ΔΔCt method. **p<0.01 and ***p<0.001 versus control(phosphate buffer -treated PC12 cells) by Student’s t-test

Our previous study indicated close relationship between ASN and APP levels. Moreover, it was found that ASN enhanced Aβ peptides secretion and its toxicity leading to irreversible alterations in cells viability [11]. In this study the effect of eASN on expression of enzymes engaged in APP metabolism and in degradation of Aβ peptides was investigated. Our results demonstrated significant downregulation of gene expression of α-secretase (*Adam10*), the key enzyme in non-amyloidogenic APP processing (Fig. 4a). eASN had no effect on gene expression of β-secretase (*Bace1*) and also did not affect γ-secretase crucial subunits, presenilin 1 and presenilin 2 (*Psen1,2*) (Fig. 4a). However, eASN decreased gene expression of *Mmp2* and *Mmp10* and upregulated gene expression of *Mmp11* (Fig. 4b).

**Fig. 5.**
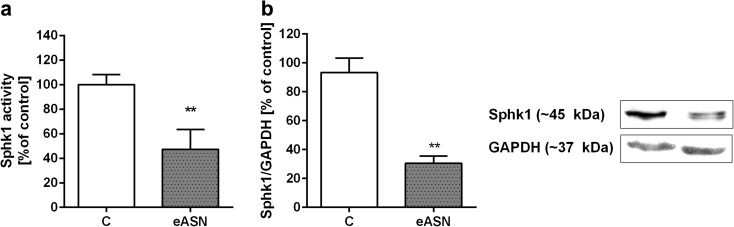
Sphk1 activity, gene expression/protein level in eASN-treated PC12 cells. PC12 cells were treated with 0,5 μM eASN for 24 h. Fluorescence value of Sphk1 activity was measured. Data represent the mean value ± S.E.M of five independent experiments **(a)**. Sphk1 (~45 kDa) immunoreactivity in the cells’ homogenate was measured. A representative Western blot from one typical experiment is shown below the graph. Data represent the mean value ± S.E.M of four independent experiments normalized against GAPDH (~37 kDa) **(b)**. *p<0.05 and **p<0.01 versus control (phosphate buffer-treated PC12 cells) by Student’s t-test.

**Fig. 6.**
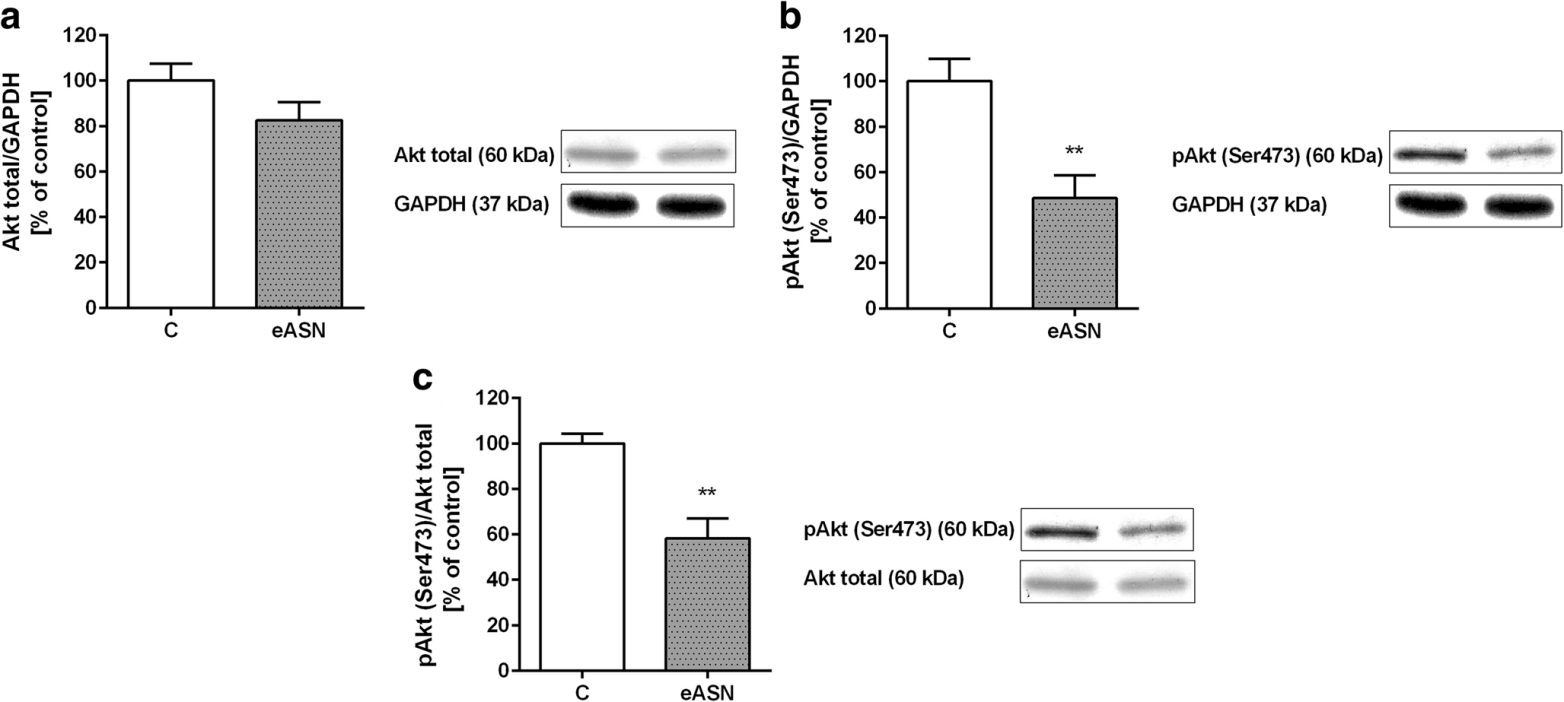
Akt kinase phosphorylation/activity under eASN toxicity. PC12 cells were treated with 0,5 μM eASN for 24 h. Effect of 0,5 μM eASN on the level of immunoreactivity of Akt (pan) **(a)**, pAkt (pSer473, ~60 kDa) **(b)** and pAkt/Akt (pan) ratio **(c)** in the cells’ homogenate. A representative Western blot from one typical experiment is shown below the graph. Data represent the mean value ± S.E.M of four independent experiments normalized against GAPDH (~37 kDa) **(a,b)**. **p<0.01 versus control (phosphate buffer -treated PC12 cells) by Student’s t-test.

**Fig. 7.**
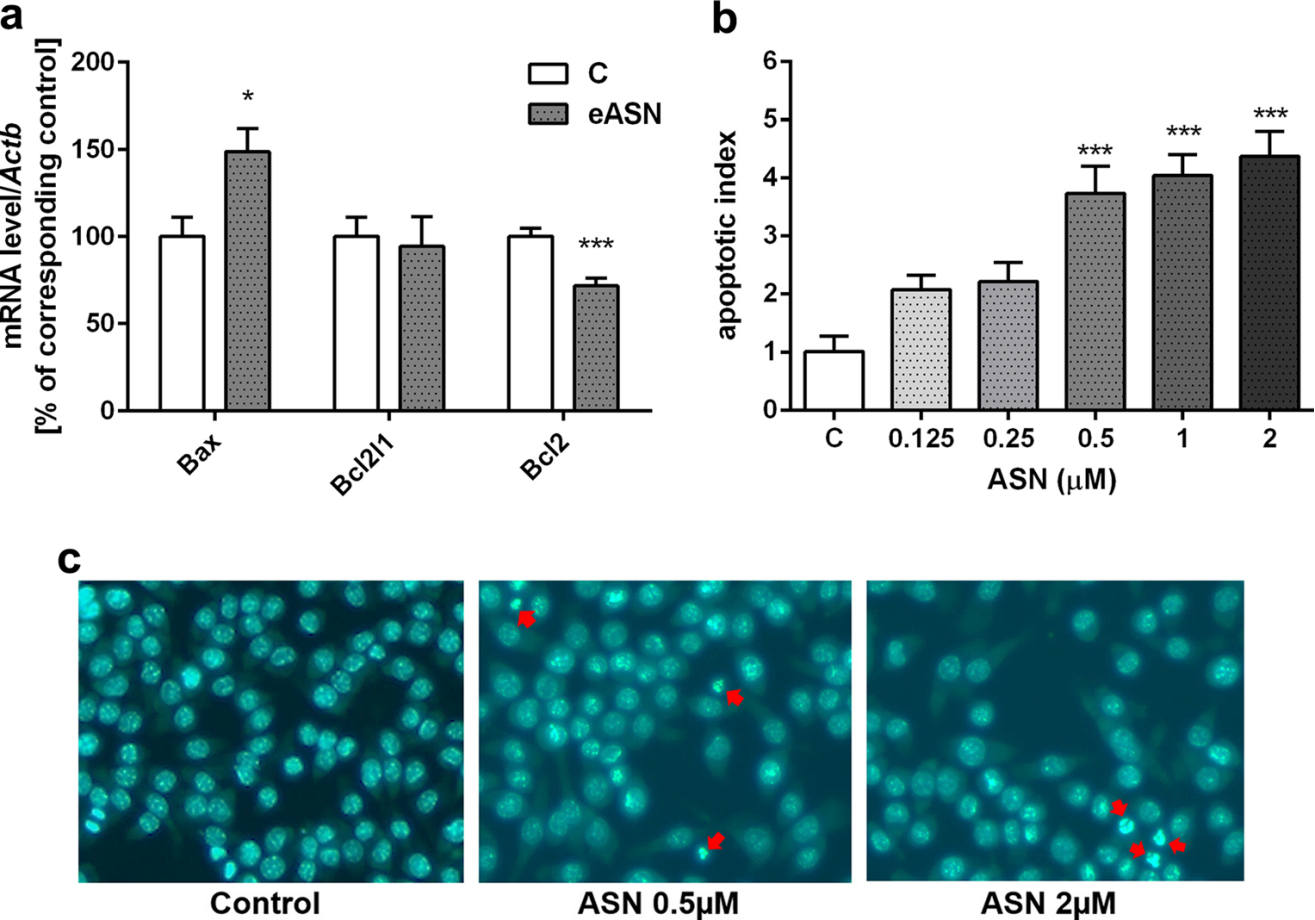
The effect of eASN on Bcl-2 pro-apoptotic and anti-apoptotic proteins gene expression and on apoptotic cells’ death. The mRNA level of *Bax, Bcl2* and *Bcl2-L1* after 24 h of 0,5 μM eASN treatment was measured with real-time PCR **(a)**. The value expresses the fold of the above gene stimulation normalized against *Actb* (β-actin). Microscopic examination of cell nuclei, stained with DNA-binding fluorochrome Hoechst 33342. The cells were treated with 0,5 μM eASN, 24h. Cells with typical apoptotic nuclear morphology (nuclear shrinkage, chromatin condensation) were identified and counted. The results were expressed as percentages of apoptotic cells in the whole cells’ population from one exemplary experiment in four to eight replications **(b,c)**. Data represent the mean value ± S.E.M of four – eight separate experiments with two replications. The relative level of mRNA was calculated by ΔΔCt method. **p<0.01 versus control(phosphate buffer -treated PC12 cells) by Student’s t-test

Other crucial enzymes involved in regulation of cell survival and death are sphingosine kinase (SphK1) and PI3K/Akt kinase. It was found that eASN induced a significant decrease in the activity and protein level of Sphk1 (Fig. 5a, b). Similar effects as eASN were exhibited by ASN-mutated forms, i.e. A30P, E46K and A53T on PC12 cells’ viability and the Sphk1 activity (Fig. S1a, b). It was observed that eASN also decreased the pro-survival pathway regulated by Akt kinase. The protein level of total Akt was not altered (Fig. 6a), but significantly lower phosphorylation of Akt kinase on serine 473 was observed, which may be responsible for its lower activity (Fig. 6b). In consequence, the ratio of phospho-Akt to total Akt was significantly lower after ASN treatment as compared to the control value (Fig. 6c). It was previously shown that Akt inhibits cells’ death by preventing the release of cytochrome c from mitochondria and by regulation of pro and anti-apoptotic Bcl-2 proteins. Our study indicated that eASN enhanced expression of the pro-apoptotic Bcl-2 protein, *Bax*, and downregulated the anti-apoptotic protein *Bcl2* (Fig. 7a). Moreover, eASN activated apoptotic cells’ death was visualised by nuclei staining (Hoechst 33342) (Fig. 7b). Representative pictures showed enhanced number of cells with typical apoptotic morphological changes in cell nuclei characterized by nuclear shrinkage, chromatin condensation and nuclear fragmentation (Fig. 7c).

Furthermore, we also analysed several compounds as S1P analogues (SEW2871, p-FTY720), the caspase inhibitor (Z-DEVD-FMK) and an inhibitor of the inner mitochondria membrane permeability (Cyclosporin A) in order to evaluate their potentially protective effect against eASN. As a result no effect of those compounds on cells’ viability was observed. Moreover, neither Resveratrol nor quercetin, specific SIRT2 inhibitor (AK-7) nor inhibitor of PARP-1 (PJ-34) were able to rescue cells against eASN toxicity (Fig. S2).

**Fig. 8.**
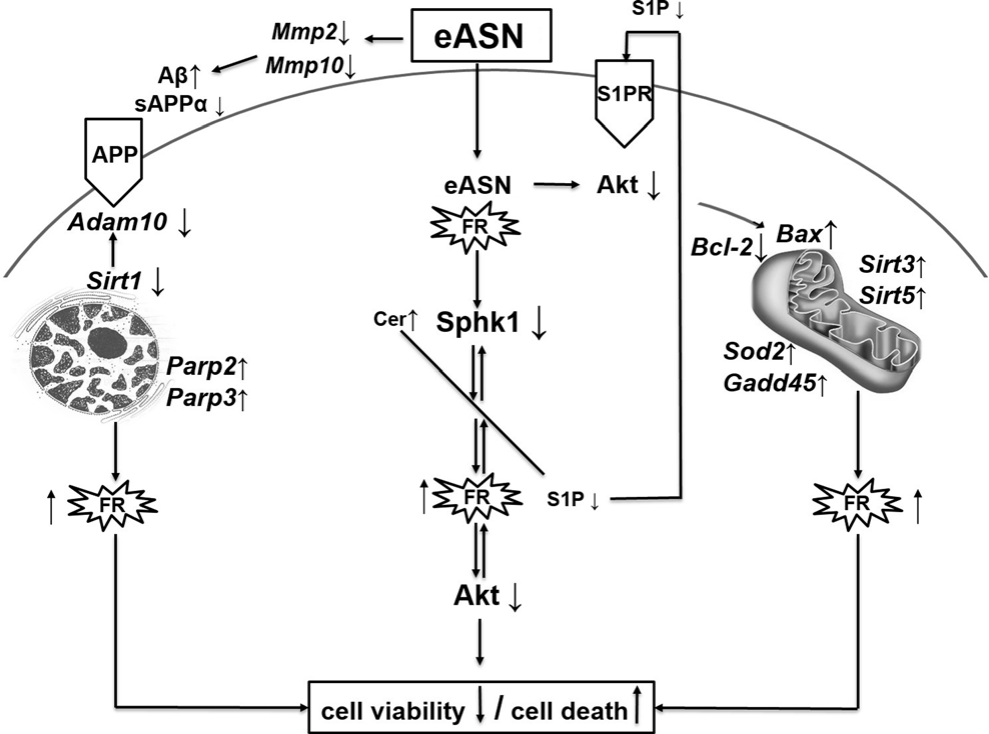
Schematic representation of eASN evoked alteration of gene expression and molecular changes leading to decrease of cells’ viability and to activation of cells’ death.

All described molecular alterations evoked by eASN were demonstrated on Fig. 8.

## Discussion

Our results showed that eASN may play an important role as a potent regulator of transcription. It differently affects gene expression of SIRT1 and mitochondrial SIRT3 and SIRT5. It was found that eASN decreased mRNA level of SIRT1. Moreover, eASN downregulated the expression of *Adam10*, the enzyme responsible for non-amyloidogenic APP metabolism. The inhibition of *Adam10* by ASN may disturb the balance between the non-amyloidogenic and amyloidogenic pathways of APP processing. Previous studies showed that SIRT1 deletion decreased lifespan and enhanced ASN aggregates in brain of PD mouse experimental model [50, 51]. The upregulation of SIRT1 leads to suppression of Aβ production by activation of alpha-secretase [51, 53]. eASN may translocate from extracellular compartment inside the cell and it can influence gene expression directly or by interaction with different transcription factors, however this process is not fully understood [66]. Additionally, it was previously found that ASN significantly upregulated the APP protein level and Aβ secretion [11, 23]. All the above-mentioned data together indicate the important relationship between ASN/APP/Aβ and suggest that ASN/Aβ interaction can lead to irreversible molecular alterations and to cell death [11]. eASN by inhibition of *Adam10* and by downregulation of gene expression of *Mmp2*, *Mmp10* with concomitant activation of *Mmp11* may alter APP/Aβ processing and may lead to higher Aβ production. It was demonstrated that MMP2 and MMP9 may be involved in the Aβ catabolism, as they can degrade Aβ fibrils *in vitro* as well as amyloid plaques in brain slices from APP/PS1 mice. MMPs were found in the brains of AD patients [67–69] and the results indicated that they participated in Aβ clearance by its degradation. Wan et al. (2015) demonstrated that Aβ-42 oligomers induced leakage of the blood-brain barrier (BBB) and that MMPs may play an important role in this process [70]. Our data demonstrated that ASN significantly decreased the transcription of *Mmp2* and *Mmp10* which may be responsible for the lower Aβ catabolism leading, in consequence, to a higher Aβ concentration. Moreover, it is possible to suggest that the upregulation of *Mmp11* may enhance APP degradation. It was previously proposed that MMP12 exacerbated the cascade of proteolytic processes by subsequent activation of several MMPs [71]. The involvement of ASN in the APP/Aβ metabolism by downregulation of *Adam10, Mmp2* and *Mmp10* expression may have a significant impact on the cells’ fate.

Moreover, ASN through the inhibition of pro-survival kinases Sphk1 and Akt could profoundly affect cells’ viability. Our results showed that both native and mutated eASN similarly decreased the activity of Sphk1. Recently, we also demonstrated that Sphk1 inhibition stimulated ASN secretion, the release of cytochrome c from the mitochondria, activated pro-apoptotic protein expression and led to caspase-dependent dopaminergic cells’ death in stress induced by MPP+ [22, 41]. Our studies suggested that Sphk1 inhibition by activation of oxidative stress led to ASN release into the extracellular compartment [22]. Previous data demonstrated the role of oxidative/nitrosative stress in ASN release from the brain synaptosomal fraction [29]. Moreover, it was found that eASN induced Aβ release and that prolonged action of ASN (10 μM for 48 h) led to cell death [11, 72]. In the present work the eASN- evoked Sphk1 decline could also be explained on the basis of the action of reactive oxygen species (ROS). Oxidative stress can regulate Sphk1 expression and activity depending on cell’s type and intensity of stress [73]. It was reported that ROS overproduction, induced by Aβ peptides and MPP^+^, may decrease Sphk1 activity in SH-SY5Y cells [41, 74, 75]. eASN via Sphk(s) inhibition disturbs the sphingolipid homeostasis, which may lead to lower S1P synthesis, and concomitantly to lower pro-survival signaling through S1P-specific receptors. A growing number of studies have emphasized the important role of bioactive sphingolipids such as S1P and ceramide in the regulation of neuronal cell survival and death, respectively. The sphingolipid equilibrium between S1P and ceramide (also called the sphingolipid rheostat) may be crucial for cell survival [38, 43, 73, 76]. Several studies have indicated that an increased ceramide concentration suppressed the viability of dopaminergic neuronal cells [43, 77, 78]. It was also shown that disturbances in the S1P level and signaling could be responsible for the pathomechanism of AD and other neurodegenerative diseases [79, 80]. We hypothesized that lower S1P synthesis may be also important in the mechanism of cell death evoked by eASN. Sphk(s) pharmacological inhibition has a similar effect as MPP+ on dopaminergic cell viability [22]. Another very important pro-survival pathway inhibited by eASN is Akt, which is also involved in S1P receptor-mediated signaling. It was demonstrated previously that ASN has a dual effects on Akt phosphorylation/activity depending on its structure and concentration [81–83]. In our study eASN significantly reduced Akt phosphorylation/activity which, in consequence, may decrease cells’ viability.

There is a strong evidence that ASN overexpression enhances the ROS level [12, 84, 85]. Our previous data showed that eASN enhanced the calcium influx by voltage-dependent calcium channels and disturbed mitochondrial function, which in consequence induced oxidative stress, altered CdK5 and GSK-3β and activated caspase-dependent programmed cell death [11, 86–88]. The production of ROS, which is well established to occur during abnormal protein conformation, e.g. ASN or Aβ peptide oligomerization, could be a common fundamental mechanism in neurodegenerative disorders such as AD and PD [12, 58, 76, 89, 90]. ASN itself or its fragment(s) directly or through the interaction with different transcription factors can influence gene expression for several important enzymes as SIRT1, α-secretase and other proteins as Bax/Bcl2. Concomitantly eASN leads to significantly higher expression of *Sod2*, which is one of the most important anti-oxidative enzymes responsible for dismutation of superoxide in the mitochondria. Additionally, higher expression of the *Gpx4* and *Gadd45b* were found*.*

Moreover, eASN up-regulates *Sirt3* and *Sirt5* expression, which may have anti-apoptotic properties [46, 91]. The data suggest that SIRT3 may be mainly engaged in the activation of an antioxidative mechanism against ASN toxicity. SIRT1 and SIRT3 play a significant role in the regulation of redox potential, energy and metabolic status of the cell. SIRT1 and SIRT3 deacetylate SOD1 and SOD2, respectively. SIRT1 exerts the effect through the regulation of α-secretase, heat shock protein (HSP) [50, 51, 92] and PGC1-α, which results in enhanced mitochondria biogenesis [47]. However, the lower expression of SIRT1 observed in this study may lead to disruption of the SIRT1–PGC1-α relationships. This alteration has been suggested as being of significance for DNA repair impairment and may play an important role in the pathomechanism of neurodegenerative diseases. The role of SIRT5 is not till now fully elucidated. This enzyme is also responsible for protein desuccinylation and demalonylation, and for cytochrome c release from mitochondria.

Lower *Sirt1* expression may affect several cellular processes, including PARP1 function. It was reported that SIRT1 has been able to mitigate rapid PARP1 activation in oxidative stress by deacetylated PARP1 reversing its enzymatic stimulation and reducing it activity to nearly undetectable levels. Moreover, SIRT1 has been shown to interact with the DNA-binding domain and, to a lesser extent, with the automodification domain of PARP1 [93]. It was also reported that PARP2 may regulate the activity of the SIRT1 [94] and SIRT1 promotor. We showed that eASN upregulated gene expression for the DNA-bound enzyme PARP2 and PARP3. The previous studies demonstrated the significance of PARP1 in neurodegenerative disorders [95]. There are evidences indicating the role of DNA-bound PARP(s) in regulation of transcription factors, DNA repair and SIRT(s). The relationship between PARP(s) and SIRT(s) may have a significant impact on cells’ fate in pathological conditions, including neurodegenerative disorders. These two families of enzymes were previously suggested to compete for the same substrate βNAD^+^. However, recently published data have demonstrated that, opposite to PARP1, the affinity of PARP2 and probably PARP3 to NAD is similar as in the case of SIRT1, and that many functions of PARP2 are independent from NAD [93, 94]. Our data indicate that eASN may serve as important modulator of transcription and may exert its toxicity through alterations of gene expression of enzymes involved in APP/AB metabolism and through inhibition of Sphk1 and Akt mediated signaling. All these data suggest that eASN may have a significant impact on the progression of neurodegenerative disorders.

## Electronic supplementary material

Fig. S1. The effect of eASN native and mutated forms on Sphk1 activity and PC12 cells’ viability. PC12 cells were treated with 0,5 μM eASN in native (eASNn) and mutated (E46K, A53T, A30P) forms for 24 h. Fluorescence value of Sphk1 activity **(a)** and cells’ viability by MTT assay **(b)** were measured. Data represent the mean value ± S.E.M of four independent experiments. *p<0.05, **p<0.01 and ***p<0.001 versus control (phosphate buffer -treated PC12 cells) by one-way ANOVA followed by the Newman–Keuls post-hoc test. (JPEG 202 kb).

(JPEG 189 kb).

Fig. S2. The effect of selected pharmacological compounds on PC-12 cells’ viability. Cells were treated for 1 h with following compounds: SEW 2871 (10 μM), FTY720-P (100 pM), cyclosporin A (CsA 2 μM), resveratrol (RSV: 0,1- 25 μM), quercetin (Q, 0,1-100 μM), AK-7 (20 μM), PJ-34 (20 μM) and then exposed to 0,5 μM eASN for 24 h. Cells’ viability was determined by MTT assay. Data represent the mean value ±S.E.M of four independent experiments with six replications. ***p<0.001 versus control (phosphate buffer -treated PC12 cells) by one-way ANOVA followed by the Newman–Keuls post-hoc test. (JPEG 243 kb).

## Abbreviations

**A30P**: α-synuclein mutated protein
**A53T**: α-synuclein mutated protein
**AD**: Alzheimer’s disease
**ADAM10 (gene name Adam10****)**: alpha-secretase
**AIF**: apoptosis-inducing factor
**APP**: amyloid precursor protein
**ASN**: alpha-synuclein
**ATP**: adenosine triphosphate
**Aβ**: amyloid beta
**BACE1 (gene name*****Bace1*****)**: beta-site amyloid precursor protein cleaving enzyme 1
**Bax (gene name*****Bax*****)**: pro-apoptotic Bcl-2 protein
**Bcl-2 (gene name*****Bcl2*****)**: anti-apoptotic Bcl-2 protein
**Bclx-L (gene name*****Bcl2l1*****)**: anti-apoptotic Bcl-2 protein
**CCCP**: carbonyl cyanide 3-chlorophenylhydrazone (mitochondrial uncoupler)
**Cyb5b (gene name*****Cyb5b*****)**: cytochrome b5
**DCF**: 2’7’-dichlorofluorescein
**DMEM**: Dulbecco's Modified Eagle Medium
**E45K**: α-synuclein mutated protein
**eASN**: extracellular acting ASN
**ETC**: electron transport protein complexes
**EDTA**: ethylenediaminetetraacetic acid
**FBS**: fetal bovine serum
**Gadd45b (gene name*****Gadd45b*****)**: anti-apoptotic protein growth arrest and DNA damage inducible beta
**GPx-4 (gene name*****Gpx4*****)**: glutathione peroxidase 4
**H2DCF-DA**: 2’,7’ dichlorodihydrofluorescein diacetate
**HSP**: heat shock protein
**LB**: Lewy body
**MMP 2, 10, 11 (gene name*****Mmp2, 10, 11*****)**: metalloproteinase 2, 10, 11
**MPP+/MPTP**: 1-methyl-4-phenylpyridinium/1-methyl-4-phenyl-1,2,3,6-tetrahydropyridine
**MSS/HPLC**: mass spectrometry/high performance liquid chromatography
**MTT**: 3-(4,5-dimethylthiazol-2-yl)-2,5-diphenyltetrazolium bromide
**NAD**: nicotinamide adenine dinucleotide
**PAR**: poly(ADP-ribose)
**PARP 1, 2, 3**: poly(ADP-ribose) polymerase 1, 2, 3
**PBS**: phosphate buffer saline
**PC12**: pheochromocytoma cell line
**PD**: Parkinson’s disease
**p-FTY720**: FTY720 Phosphate, 2-amino-2[2-(4-octylphenyl)ethyl]-1,3-propanediol, mono dihydrogen phosphate ester
**PGC1**α: Peroxisome proliferator-activated receptor (PPAR)γ coactivator 1α
**PI3K/Akt**: phosphatidylinositol-3-kinase/Akt
**PJ-34**: N-(6-Oxo-5,6-dihydrophenanthridin-2-yl)-(N,N-dimethylamino)acetamide hydrochloride, specific PARP inhibitor
**Psen1, 2 (gene name*****Psen1, 2*****)**: presenilin 1,2
**ROS**: reactive oxygen species
**S1P**: sphingosine-1-phosphate
**SDS-PAGE**: sodium dodecyl sulfate polyacrylamide gel electrophoresis
**SEW2871**: S1P_1_ receptor agonist
**SIRT 1, 2, 3, 4, 5 (gene name*****Sirt1, 2, 3, 4, 5***): sirtuin 1, 2, 3, 4, 5
**SOD2 (gene name*****Sod2*****)**: superoxide dismutase 2
**Sphk1**: sphingosine kinase 1

## References

[1] Stefanis L (2012) a -Synuclein in Parkinson’ s Disease. Cold Spring Harb Perspect Med 1–23. doi: 10.1101/cshperspect.a009399 10.1101/cshperspect.a009399 PMC3281589 22355802

[2] Cookson M (2009). alpha-Synuclein and neuronal cell death. Mol Neurodegener.

[3] Dehay B, Decressac M, Bourdenx M (2016). Targeting α-synuclein: therapeutic options. Mov Disord.

[4] Vila M, Vukosavic S, Jackson-Lewis V (2000). Alpha-synuclein up-regulation in substantia nigra dopaminergic neurons following administration of the parkinsonian toxin MPTP. J Neurochem.

[5] Mattila PM, Rinne JO, Helenius H (2000). Alpha-synuclein-immunoreactive cortical Lewy bodies are associated with cognitive impairment in Parkinson’s disease. Acta Neuropathol.

[6] Auluck PK (2002). Chaperone suppression of alpha -synuclein toxicity in a drosophila model for Parkinson’s disease. Science.

[7] Fujiwara H, Hasegawa M, Dohmae N (2002). alpha-Synuclein is phosphorylated in synucleinopathy lesions. Nat Cell Biol.

[8] Bartels T, Choi JG, Selkoe DJ (2011). α-Synuclein occurs physiologically as a helically folded tetramer that resists aggregation. Nature.

[9] Wang W, Perovic I, Chittuluru J (2011). A soluble α-synuclein construct forms a dynamic tetramer. Proc Natl Acad Sci U S A.

[10] Mikolaenko I, Pletnikova O, Kawas CH (2005). Alpha-synuclein lesions in normal aging, Parkinson disease, and Alzheimer disease: evidence from the Baltimore Longitudinal Study of Aging (BLSA). J Neuropathol Exp Neurol.

[11] Kazmierczak A, Strosznajder JB, Adamczyk A (2008). α-Synuclein enhances secretion and toxicity of amyloid beta peptides in PC12 cells. Neurochem Int.

[12] Wilkaniec A, Strosznajder JB, Adamczyk A (2013). Toxicity of extracellular secreted alphasynuclein: Its role in nitrosative stress and neurodegeneration. Neurochem Int.

[13] Ryan BJ, Hoek S, Fon EA, Wade-Martins R (2015). Mitochondrial dysfunction and mitophagy in Parkinson's: from familial to sporadic disease. Trends Biochem Sci..

[14] Lashuel HA, Petre BM, Wall J (2002). α-synuclein, especially the parkinson’s diseaseassociated mutants, forms pore-like annular and tubular protofibrils. J Mol Biol.

[15] Uversky VN, Li J, Fink AL (2001). Evidence for a partially folded intermediate in alpha- synuclein fibril formation. J Biol Chem.

[16] Winner B, Jappelli R, Maji SK (2011). In vivo demonstration that alpha-synuclein oligomers are toxic. Proc Natl Acad Sci U S A.

[17] Spillantini MG, Schmidt ML, Lee VM (1997). Alpha-synuclein in Lewy bodies. Nature.

[18] Spillantini MG, Crowther RA, Jakes R (1998). Synuclein in filamentous inclusions of Lewy bodies from Parkinson’s disease and dementia with Lewy bodies. Proc Natl Acad Sci.

[19] Galvin JE, Uryu K, Lee VM, Trojanowski JQ (1999). Axon pathology in Parkinson’s disease and Lewy body dementia hippocampus contains alpha-, beta-, and gamma-synuclein. Proc Natl Acad Sci U S A.

[20] Braak H, Ghebremedhin E, Rüb U (2004). Stages in the development of Parkinson’s disease-related pathology. Cell Tissue Res.

[21] Cookson MR (2005). The biochemistry of Parkinson’s disease. Annu Rev Biochem.

[22] Pyszko JA, Strosznajder JB (2014). The key role of sphingosine kinases in the molecular mechanism of neuronal cell survival and death in an experimental model of Parkinson’s disease. Folia Neuropathol.

[23] Jesko H, Okada T, Strosznajder RP, Nakamura S (2014). Sphingosine kinases modulate the secretion of amyloid β precursor protein from SH-SY5Y neuroblastoma cells: the role of α-synuclein. Folia Neuropathol.

[24] Surguchev A, Surguchov A (2015). Effect of alpha-synuclein on membrane permeability and synaptic transmission: a clue to neurodegeneration?. J Neurochem.

[25] Pacheco CR, Morales CN, Ramírez AE (2015). Extracellular alpha-synuclein alters synaptic transmission in brain neurons by perforating the neuronal plasma membrane. JNeurochem.

[26] Desplats P, Lee H-J, Bae E-J (2009). Inclusion formation and neuronal cell death through neuron-to-neuron transmission of alpha-synuclein. Proc Natl Acad Sci U S A.

[27] Luk KC, Song C, O’Brien P (2009). Exogenous alpha-synuclein fibrils seed the formation of Lewy body-like intracellular inclusions in cultured cells. Proc Natl Acad Sci U S A.

[28] Ouzounoglou E, Kalamatianos D, Emmanouilidou E (2014). In silico modeling of the effects of alpha-synuclein oligomerization on dopaminergic neuronal homeostasis. BMC Syst Biol.

[29] Adamczyk A, Czapski GA, Kaźmierczak A, Strosznajder JB (2009). Effect of N-methyl-Daspartate (NMDA) receptor antagonists on alpha-synuclein-evoked neuronal nitric oxide synthase activation in the rat brain. Pharmacol Rep.

[30] Lee H-J, Bae E-J, Lee S-J (2014). Extracellular α--synuclein-a novel and crucial factor in Lewy body diseases. Nat Rev Neurol.

[31] Lee HJ, Suk JE, Patrick C (2010). Direct transfer of alpha-synuclein from neuron to astroglia causes inflammatory responses in synucleinopathies. J Biol Chem.

[32] Emmanouilidou E, Elenis D, Papasilekas T (2011). Assessment of a-synuclein secretion in mouse and human brain parenchyma. PLoS One.

[33] Danzer KM, Haasen D, Karow AR (2007). Different species of alpha-synuclein oligomers induce calcium influx and seeding. J Neurosci.

[34] Danzer KM, Krebs SK, Wolff M (2009). Seeding induced by alpha-synuclein oligomers provides evidence for spreading of alpha-synuclein pathology. J Neurochem.

[35] Nonaka T, Watanabe ST, Iwatsubo T, Hasegawa M (2010). Seeded aggregation and toxicity of {alpha}-synuclein and tau: cellular models of neurodegenerative diseases. J Biol Chem.

[36] Hansen C, Angot E, Bergström AL (2011). α-Synuclein propagates from mouse brain to grafted dopaminergic neurons and seeds aggregation in cultured human cells. J Clin Invest.

[37] Mizugishi K, Yamashita T, Olivera A (2005). Essential role for sphingosine kinases in neural and vascular development essential role for sphingosine kinases in neural and vascular development. Mol Cell Biol.

[38] Cuvillier O, Pirianov G, Kleuser B (1996). Suppression of ceramide-mediated programmed cell death by sphingosine-1-phosphate. Nature.

[39] Kanno T, Nishizaki T, Proia RL (2010). Regulation of synaptic strength by sphingosine 1-phosphate in the hippocampus. Neuroscience.

[40] Couttas TA, Kain N, Daniels B (2014). Loss of the neuroprotective factor sphingosine 1- phosphate early in Alzheimer’s disease pathogenesis. Acta Neuropathol Commun.

[41] Pyszko J, Strosznajder JB (2014). Sphingosine kinase 1 and sphingosine-1-phosphate in oxidative stress evoked by 1-methyl-4-phenylpyridinium (MPP+) in human dopaminergic neuronal cells. Mol Neurobiol.

[42] Czubowicz K, Cieślik M, Pyszko J (2015). Sphingosine-1-phosphate and its effect on glucose deprivation/glucose reload stress: from gene expression to neuronal survival. Mol Neurobiol.

[43] Czubowicz K, Strosznajder R (2014) Ceramide in the molecular mechanisms of neuronal cell death. The role of sphingosine-1-phosphate. Mol Neurobiol 1–12. doi: 10.1007/s12035-013-8606-4 10.1007/s12035-013-8606-4 PMC4181317 24420784

[44] Ceccom J, Loukh N, Lauwers-Cances V (2014). Reduced sphingosine kinase-1 and enhanced sphingosine 1-phosphate lyase expression demonstrate deregulated sphingosine 1-phosphate signaling in Alzheimer’s disease. Acta Neuropathol Commun.

[45] Sivasubramanian M, Kanagaraj N, Dheen ST, Tay SSW (2015). Sphingosine kinase 2 and sphingosine-1-phosphate promotes mitochondrial function in dopaminergic neurons of mouse model of Parkinson’s disease and in MPP+ −treated MN9D cells in vitro. Neurosci.

[46] Kincaid B, Bossy-Wetzel E (2013). Forever young: SIRT3 a shield against mitochondrial meltdown, aging, and neurodegeneration. Front Aging Neurosci.

[47] Lavu S, Boss O, Elliott PJ, Lambert PD (2008). Sirtuins--novel therapeutic targets to treat ageassociated diseases. Nat Rev Drug Discov.

[48] Kim HS, Vassilopoulos A, Wang RH (2011). SIRT2 maintains genome integrity and suppresses tumorigenesis through regulating APC/C activity. Cancer Cell.

[49] Zhong L, Mostoslavsky R (2011). Fine tuning our cellular factories: sirtuins in mitochondrial biology. Cell Metab.

[50] Guo YJ, Dong SY, Cui XX, Feng Y, Liu T, Yin M, Kuo SH, Tan EK (2016). Resveratrol alleviates MPTP-induced motor impairments and pathological changes by autophagic degradation of α-synuclein via SIRT1-deacetylated LC3. Mol Nutr Food Res..

[51] Ng F, Wijaya L, Tang BL (2015). SIRT1 in the brain-connections with aging-associated disorders and lifespan. Front Cell Neurosci..

[52] Min SW, Cho SH, Zhou Y (2010). Acetylation of tau inhibits its degradation and contributes to tauopathy. Neuron.

[53] Wang XF, Liu DX, Liang Y, Xing LL, Zhao WH, Qin XX, Shang DS, Li B (2016). Cystatin C shifts APP processing from amyloid-β production towards non-amyloidgenic pathway in brain endothelial cells. PLoS One..

[54] Postina R (2008). A closer look at alpha-secretase. Curr Alzheimer Res.

[55] Outeiro TF, Kontopoulos E, Altmann SM (2007). Sirtuin 2 inhibitors rescue alphasynuclein-mediated toxicity in models of Parkinson’s disease. Science.

[56] Mendoza-Alvarez H, Alvarez-Gonzalez R (1993). Poly(ADP-ribose) polymerase is a catalytic dimer and the automodification reaction is intermolecular. J Biol Chem.

[57] Houtkooper RH, Cantó C, Wanders RJ, Auwerx J (2010). The secret life of NAD+: an old metabolite controlling new metabolic signaling pathways. Endocr Rev.

[58] Strosznajder RP, Czubowicz K, Jesko H, Strosznajder JB (2010). Poly(ADP-ribose) metabolism in brain and its role in ischemia pathology. Mol Neurobiol.

[59] Pieper AA, Blackshaw S, Clements EE (2000). Poly(ADP-ribosyl)ation basally activated by DNA strand breaks reflects glutamate-nitric oxide neurotransmission. Proc Natl Acad Sci U S A.

[60] Langelier MF, Riccio AA, Pascal JM (2014). PARP-2 and PARP-3 are selectively activated by 5’ phosphorylated DNA breaks through an allosteric regulatory mechanism shared with PARP-1. Nucleic Acids Res.

[61] Yu S-W, Andrabi SA, Wang H (2006). Apoptosis-inducing factor mediates poly(ADPribose) (PAR) polymer-induced cell death. Proc Natl Acad Sci U S A.

[62] Moroni F (2008). Poly(ADP-ribose)polymerase 1 (PARP-1) and postischemic brain damage. Curr Opin Pharmacol.

[63] Cieślik M, Czapski GA, Strosznajder JB (2015). The molecular mechanism of amyloid β42 peptide toxicity: the role of sphingosine kinase-1 and mitochondrial sirtuins. PLoS One.

[64] Don AS, Martinez-Lamenca C, Webb WR (2007). Essential requirement for sphingosine kinase 2 in a sphingolipid apoptosis pathway activated by FTY720 analogues. J Biol Chem.

[65] Lowry OH, Rosebrough NJ, Farr Al RR (1951). Protein measurement with the Folin phenol reagent. J Biol Chem.

[66] Iwata A, Miura S, Kanazawa I (2001). alpha-Synuclein forms a complex with transcription factor Elk-1. J Neurochem.

[67] Yan P, Hu X, Song H (2006). Matrix metalloproteinase-9 degrades amyloid-beta fibrils in vitro and compact plaques in situ. J Biol Chem.

[68] Backstrom JR, Lim GP, Cullen MJ, Tökés ZA (1996). Matrix metalloproteinase-9 (MMP-9) is synthesized in neurons of the human hippocampus and is capable of degrading the amyloidbeta peptide (1–40). J Neurosci.

[69] Brkic M, Balusu S, Libert C, Vandenbroucke RE (2015). Friends or foes: matrix metalloproteinases and their multifaceted roles in neurodegenerative diseases. Mediators Inflamm.

[70] Wan W, Cao L, Liu L (2015). Aβ(1–42) oligomer-induced leakage in an in vitro bloodbrain barrier model is associated with up-regulation of RAGE and metalloproteinases, and down-regulation of tight junction scaffold proteins. J Neurochem.

[71] Ito S, Kimura K, Haneda M (2007). Induction of matrix metalloproteinases (MMP3, MMP12 and MMP13) expression in the microglia by amyloid-beta stimulation via the PI3K/Akt pathway. Exp Gerontol.

[72] Adamczyk A, Kaźmierczak A (2009). Alpha-synuclein inhibits poly (ADP-ribose) polymerase- 1 (PARP-1) activity via NO-dependent pathway. Folia Neuropathol.

[73] Van Brocklyn JR, Williams JB (2012). The control of the balance between ceramide and sphingosine-1-phosphate by sphingosine kinase: Oxidative stress and the seesaw of cell survival and death. Comp Biochem Physiol - B Biochem Mol Biol.

[74] Gomez-Brouchet A, Pchejetski D, Brizuela L (2007). Critical role for sphingosine kinase-1 in regulating survival of neuroblastoma cells exposed to amyloid-beta peptide. Mol Pharmacol.

[75] Pchejetski D, Kunduzova O, Dayon A (2007). Oxidative stress-dependent sphingosine kinase-1 inhibition mediates monoamine oxidase A-associated cardiac cell apoptosis. Circ Res.

[76] Orr Gandy KA, Obeid LM (2013). Targeting the sphingosine kinase/sphingosine 1-phosphate pathway in disease: review of sphingosine kinase inhibitors. Biochim Biophys Acta - Mol Cell Biol Lipids.

[77] Martinez TN, Chen X, Bandyopadhyay S (2012). Ceramide sphingolipid signaling mediates Tumor Necrosis Factor (TNF)-dependent toxicity via caspase signaling in dopaminergic neurons. Mol Neurodegener.

[78] Sofic E, Denisova N, Youdim K (2001). Antioxidant and pro-oxidant capacity of catecholamines and related compounds. Effects of hydrogen peroxide on glutathione and sphingomyelinase activity in pheochromocytoma PC12 cells: potential relevance to age-related diseases. J Neural Transm.

[79] Thuy AV, Reimann C-M, Hemdan NY, Gräler MH (2014). Sphingosine 1-phosphate in blood: function, metabolism, and fate. Cell Physiol Biochem.

[80] Yadav RS, Tiwari NK (2014) Lipid integration in neurodegeneration: an overview of Alzheimer’s disease. Mol Neurobiol 1–9. doi: 10.1007/s12035-014-8661-5 10.1007/s12035-014-8661-5 24590317

[81] Thomas B, Mandir AS, West N (2011). Resistance to MPTP-Neurotoxicity in alphasynuclein knockout mice is complemented by human alpha-synuclein and associated with increased beta-synuclein and Akt activation. PLoS One.

[82] Kim JY, Jeon BS, Kim HJ, Ahn TB (2013). Nanomolar concentration of alpha-synuclein enhances dopaminergic neuronal survival via Akt pathway. Neural Regen Res.

[83] Seo JH, Rah JC, Choi SH (2002). Alpha-synuclein regulates neuronal survival via Bcl-2 family expression and PI3/Akt kinase pathway. FASEB J.

[84] Liu H, Sugiura M, Nava VE (2000). Molecular cloning and functional characterization of a novel mammalian sphingosine kinase type 2 isoform. J Biol Chem.

[85] Clark J, Clore EL, Zheng K (2010). Oral N-Acetyl-cysteine attenuates loss of dopaminergic terminals in α-synuclein overexpressing mice. PLoS One.

[86] Adamczyk A, Strosznajder JB (2006). Alpha-synuclein potentiates Ca2+ influx through voltage-dependent Ca2+ channels. Neuroreport.

[87] Gassowska M, Cieślik M, Wilkaniec A, Strosznajder JB (2014). Sphingosine kinases/sphingosine-1-phosphate and death signalling in APP-transfected cells. Neurochem Res.

[88] Czapski GA, Gassowska M, Wilkaniec A (2013). Extracellular alpha-synuclein induces calpain-dependent overactivation of cyclin-dependent kinase 5 in vitro. FEBS Lett.

[89] Tabner BJ, Turnbull S, El-Agnaf O, Allsop D (2001). Production of reactive oxygen species from aggregating proteins implicated in Alzheimer’s disease, Parkinson's disease and other neurodegenerative diseases. Curr Top Med Chem.

[90] Turnbull S, Tabner BJ, El-Agnaf OM (2001). α-synuclein implicated in Parkinson’s disease catalyses the formation of hydrogen peroxide in vitro. Free Radic Biol Med.

[91] Salvador JM, Brown-Clay JD, Fornace AJ (2013). Gadd45 in stress signaling, cell cycle control, and apoptosis. Adv Exp Med Biol.

[92] Watanabe S, Ageta-Ishihara N, Nagatsu S (2014). SIRT1 overexpression ameliorates a mouse model of SOD1-linked amyotrophic lateral sclerosis via HSF1/HSP70i chaperone system. Mol Brain.

[93] Bai P, Cantó C, Oudart H (2011). PARP-1 inhibition increases mitochondrial metabolism through SIRT1 activation. Cell Metab.

[94] Bai P, Canto C, Brunyánszki A (2011). PARP-2 regulates SIRT1 expression and whole body energy expenditure. Cell Metab.

[95] Strosznajder JB, Czapski GA, Adamczyk A, Strosznajder RP (2012). Poly(ADP-ribose) polymerase-1 in amyloid beta toxicity and Alzheimer’s disease. Mol Neurobiol.

