# Supplementary figures and images for: Alpha-synuclein alters differently gene expression of Sirts, PARPs and other stress response proteins: implications for neurodegenerative disorders
Source: Mol Neurobiol. 2017 Jan 3;55(1):727–40. doi: 10.1007/s12035-016-0317-1 (PMC5808059; doi:10.1007/s12035-016-0317-1)

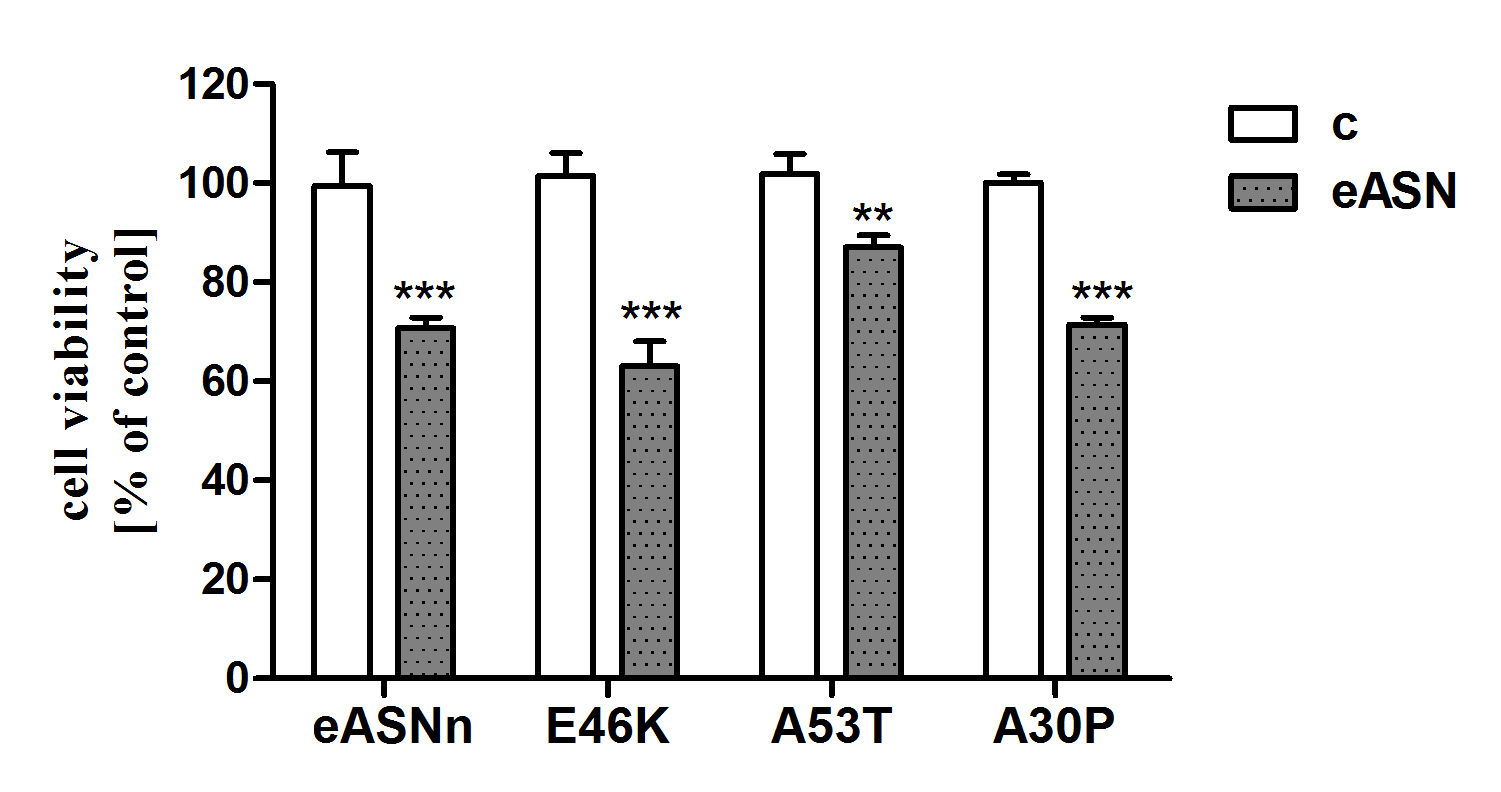

Supplement: Supplementary file 1 — The effect of eASN native and mutated forms on Sphk1 activity and PC12 cells’ viability. PC12 cells were treated with 0,5 μM eASN in native (eASNn) and mutated (E46K, A53T, A30P) forms for 24 h. Fluorescence value of Sphk1 activity (a) and cells’ viability by MTT assay (b) were measured. Data represent the mean value ± S.E.M of four independent experiments. *p<0.05, **p<0.01 and ***p<0.001 versus control (phosphate buffer -treated PC12 cells) by one-way ANOVA followed by the Newman–Keuls post-hoc test. (JPEG 202 kb). [file 12035_2016_317_Fig9_ESM.jpg]

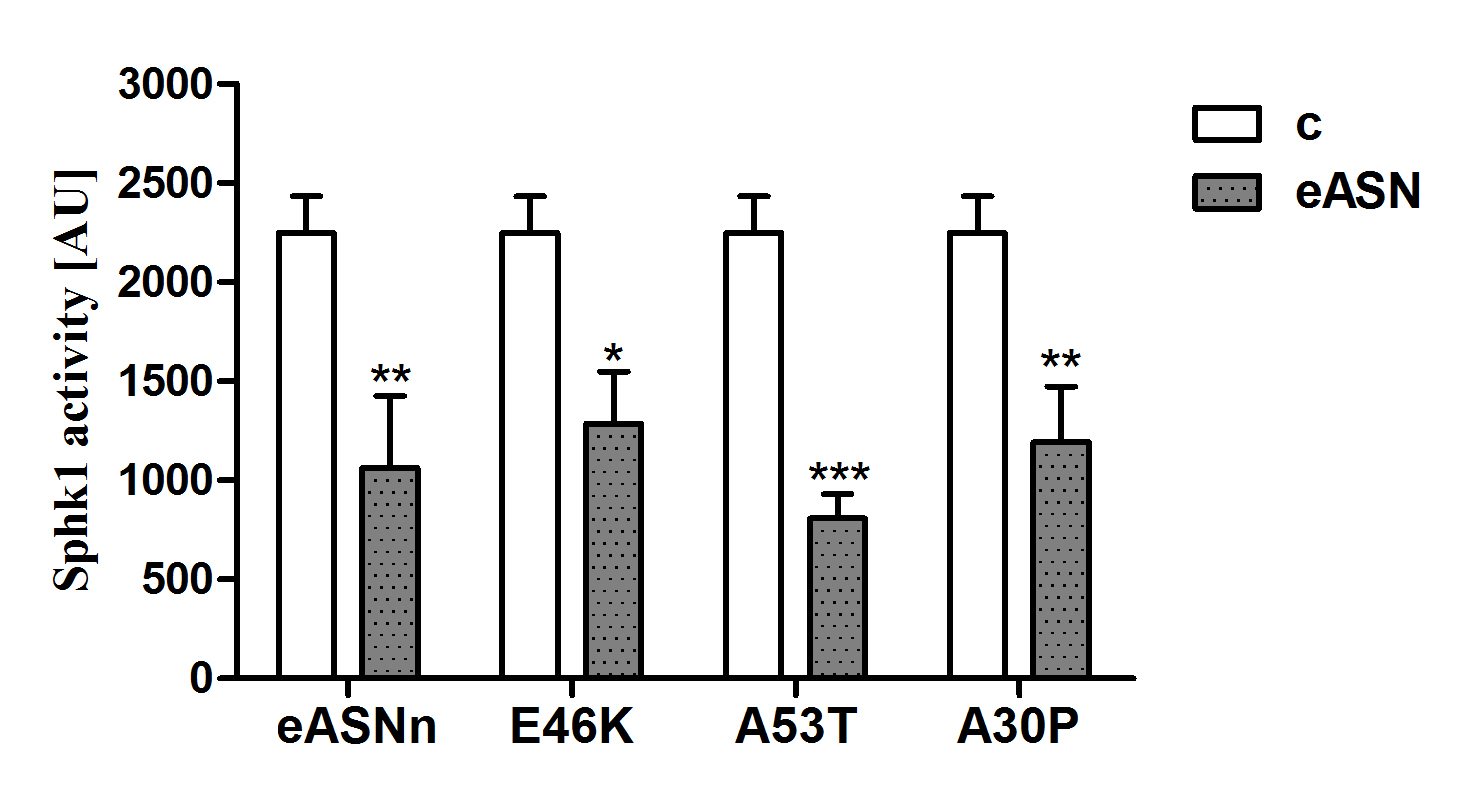

Supplement: Supplementary file 2 — (JPEG 189 kb). [file 12035_2016_317_Fig10_ESM.jpg]

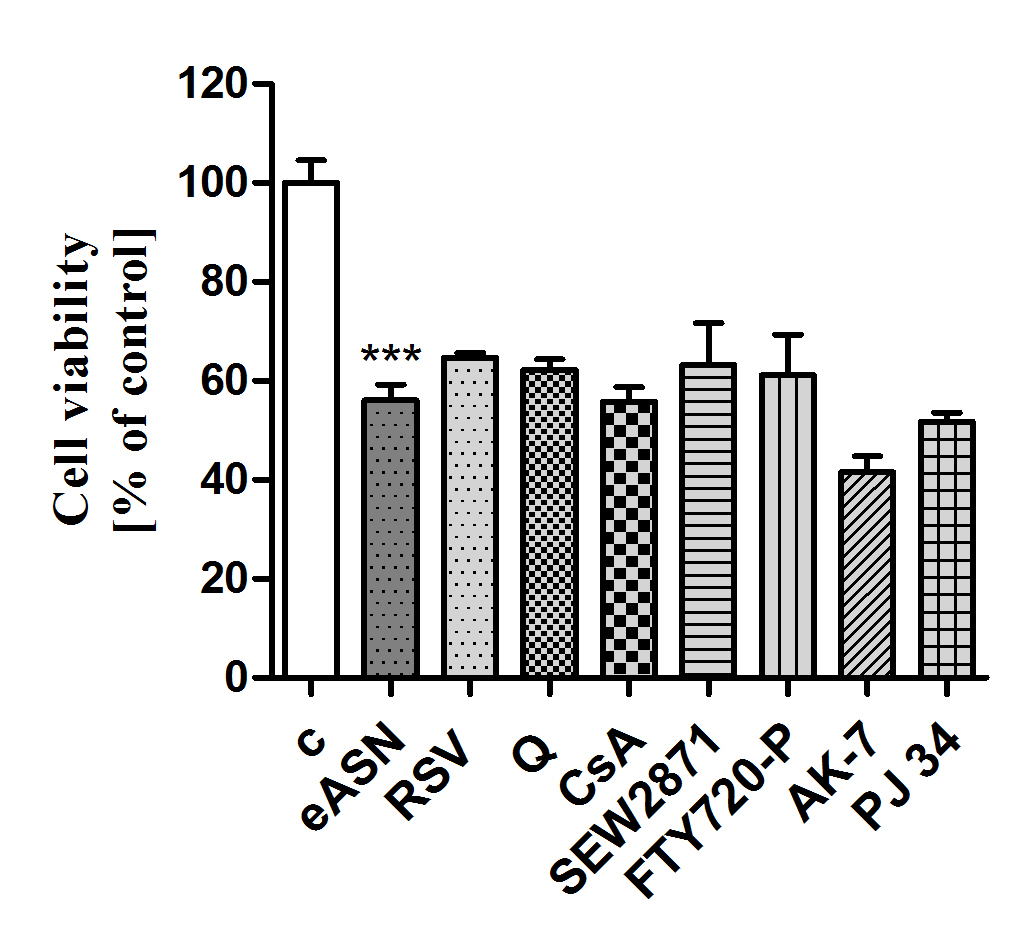

Supplement: Supplementary file 3 — The effect of selected pharmacological compounds on PC-12 cells’ viability. Cells were treated for 1 h with following compounds: SEW 2871 (10 μM), FTY720-P (100 pM), cyclosporin A (CsA 2 μM), resveratrol (RSV: 0,1- 25 μM), quercetin (Q, 0,1-100 μM), AK-7 (20 μM), PJ-34 (20 μM) and then exposed to 0,5 μM eASN for 24 h. Cells’ viability was determined by MTT assay. Data represent the mean value ±S.E.M of four independent experiments with six replications. ***p<0.001 versus control (phosphate buffer -treated PC12 cells) by one-way ANOVA followed by the Newman–Keuls post-hoc test. (JPEG 243 kb). [file 12035_2016_317_Fig11_ESM.jpg]
